# Regulation of Expression of Oxacillin-Inducible Methionine Sulfoxide Reductases in *Staphylococcus aureus*

**DOI:** 10.1155/2015/617925

**Published:** 2015-09-21

**Authors:** Kyle R. Baum, Zulfiqar Ahmad, Vineet K. Singh

**Affiliations:** ^1^Department of Microbiology and Immunology, Kirksville College of Osteopathic Medicine, A.T. Still University of Health Sciences, Kirksville, MO 63501, USA; ^2^Department of Biochemistry, Kirksville College of Osteopathic Medicine, A.T. Still University of Health Sciences, Kirksville, MO 63501, USA

## Abstract

Cell wall-active antibiotics cause induction of a locus that leads to elevated synthesis of two methionine sulfoxide reductases (MsrA1 and MsrB) in *Staphylococcus aureus*. To understand the regulation of this locus, reporter strains were constructed by integrating a DNA fragment consisting of the *msrA1/msrB* promoter in front of a promoterless *lacZ* gene in the chromosome of wild-type and MsrA1-, MsrB-, MsrA1/MsrB-, and SigB-deficient methicillin-sensitive *S. aureus* strain SH1000 and methicillin-resistant *S. aureus* strain COL. These reporter strains were cultured in TSB and the cellular levels of *β*-galactosidase activity in these cultures were assayed during different growth phases. *β*-galactosidase activity assays demonstrated that the lack of MsrA1, MsrB, and SigB upregulated the *msrA1/msrB* promoter in *S. aureus* strain SH1000. In *S. aureus* strain COL, the highest level of *β*-galactosidase activity was observed under the conditions when both MsrA1 and MsrB proteins were absent. The data suggest that the *msrA1/msrB* locus, in part, is negatively regulated by MsrA1, MsrB, and SigB in *S. aureus*.

## 1. Introduction

*Staphylococcus aureus* is part of the microbiome of roughly 30% of people who show no clinical symptoms [1]. It is an opportunistic human pathogen that can cause a wide variety of diseases and can involve any organ system in the human body. Diseases caused by* S. aureus* may include mild skin infections such as folliculitis and impetigo to fatal conditions such as pneumonia, osteomyelitis, and endocarditis [2]. Treatment of* S. aureus* infections has become problematic as it has developed numerous mechanisms to become resistant to almost all known antibiotics [3, 4].

It was previously reported that exposure of* S. aureus* to oxacillin and other cell wall-active antibiotics increases the expression of* msrA1* and* msrB* both at the transcriptional and at the protein level [5, 6]. Pathogenic bacterial species are exposed to a variety of extremely potent reactive oxygen species (ROS) by the host phagocytic cells during the course of phagocytosis that are damaging to all cellular macromolecules. ROS can cause damage to proteins by the oxidation of sulfhydryl groups, reduction of disulfides, oxidative adduction of amino acid residues close to metal-binding sites, and peptide fragmentation [7]. In particular, ROS oxidize the sulfur atom of protein-bound methionine residues resulting in methionine sulfoxide (*Met*O) and loss of protein function. However, almost all biological species possess the ability to reduce oxidized methionines [8]. MsrA and MsrB proteins reduce S- and R-epimers of methionine sulfoxides (*Met*O), respectively [8].

In* S. aureus*, genes encoding MsrA1 and MsrB are the first and second genes of a four-gene cluster that are cotranscribed [6]. A mutation in the* msrA1* gene increased the susceptibility of* S. aureus* to oxidative stress [6, 9]. More recently, it was shown that the MsrA1 protein was critical for* S. aureus* in establishing an infection in mice [10]. Interestingly, the MsrA1-deficient* S. aureus* was shown to possess an elevated level of MsrB [9] giving rise to the speculation of autoregulation of the* msrA1*/*msrB* locus. Additionally, sigma factor B (SigB) is an alternative sigma factor that is involved in regulating the expression of stress response genes in* S. aureus* [11]. Thus, it seems plausible that SigB may have a role in the regulation of the* msrA1/msrB* locus. Findings of this study provide evidence that the* msrA1/msrB* locus is negatively regulated by the products of this locus and SigB.

## 2. Materials and Methods

### 2.1. Bacterial Strains, Antibiotics, and Growth Conditions

The bacterial strains used in this study are shown in Table 1.* S. aureus* cultures were grown aerobically at 37°C in tryptic soy broth (TSB) in a shaking incubator (220 rpm) or on tryptic soy agar (TSA) by incubation for 24–48 h. Overnight cultures of* S. aureus* reporter strains were prepared in the presence of erythromycin at 10 *μ*g mL^−1^. Oligonucleotide primers used in this study were obtained from Eurofins and are shown in Table 2.

### 2.2. Transduction of* msrA1/msrB* Promoter-*lacZ* into* S. aureus* Strains

Construction of* msrA1/msrB* promoter-*lacZ* reporter strain has been previously described [6]. In this construct, a 1.3 kb DNA fragment starting 44 nucleotides downstream and going upstream of the* msrA1* gene cloned in front of a promoterless* lacZ* gene in the vector pAZ106 [12] was integrated in the chromosome of* S. aureus* strain RN450 [5, 6]. The* msrA1/msrB* promoter-*lacZ* reporter was transduced into various strains of* S. aureus* using a phage 80*α* transduction procedure. Strains used in this study were verified by PCR.

### 2.3. Determination of the* msrA1/msrB* Promoter Strength in* S. aureus*

To determine if the* msrA1/msrB* locus is autoregulated, the expression of* lacZ* from the* msrA1/msrB* promoter-*lacZ* fusion was investigated in MsrA1-, MsrB-, and MsrA1-MsrB-deficient strains of* S. aureus* strains SH1000 and COL. In addition, SigB is a major regulator of stress response in* S. aureus*. Therefore, the strength of the* msrA1/msrB* promoter was also assessed in a* sigB* mutant. Overnight cultures of these strains were diluted (1 : 100) and grown at 37°C with shaking. These cultures were grown to OD_600_ = 0.5 that was considered time 0 and the levels of *β*-galactosidase activity in these cultures were measured at different time points (0, 90, 180, 270, and 360 min) as an indicator of the strength of the* msrA1/msrB* promoter.

### 2.4. Expression of* msrA1/msrB* Promoter in the Presence of a Cell Wall-Active Antibiotic, Oxacillin

Previous studies [5, 6, 9, 10, 13] have shown that, in the presence of oxacillin, there is an increased production of MsrA1 and MsrB in* S. aureus*. To further investigate the regulation of the* msrA1/msrB* locus and to see if it can be magnified in the presence of oxacillin, overnight cultures of wild-type and the derivative* msrA1-msrB* mutant of* S. aureus* strain COL were diluted (1 : 100) in fresh TSB and grown to OD_600_ of 0.5. 10.0 mL of the culture was split into two 15 mL tubes. To one of the cultures, oxacillin was added to the final concentration of 1.0 mg mL^−1^. Both cultures with and without oxacillin were allowed to grow for an additional 2 h at 37°C with shaking. Bacterial cells were harvested by centrifugation and *β*-galactosidase activities in these cells were measured.

### 2.5. Measurement of *β*-Galactosidase Activity

The OD_600_ of the culture was determined as a measure of cell density and cells were subsequently collected by centrifugation. For precise optical density readings, cultures were diluted appropriately to bring density into measurable range. The cell pellet was used to measure *β*-galactosidase activity as described previously using O-nitrophenyl-*β*-D-galactopyranoside (ONPG) as the substrate [5, 6, 13].

### 2.6. Quantitative Real-Time PCR (qRT-PCR) Assays

qRT-PCR assays were used to verify induced expression of the genes of the* msrA1/msrB* locus under oxacillin stress and to validate the* lacZ* reporter expression data in* sigB* mutants. Cultures of* S. aureus* strain COL were grown to OD_600_ = 0.3 and divided into two tubes. One tube was stressed with oxacillin at a concentration of 1.0 mg mL^−1^ for 2 h. Total RNA was extracted from these oxacillin stressed and control cultures as described previously [14]. For the validation of* lacZ* data, the wild-type and* sigB* mutant strains of* S. aureus* were allowed to grow for 90 min and 6 h after reaching the OD_600_ = 0.5 and total RNA from these cultures were extracted. cDNA from DNase treated 0.5 *μ*g of total RNA was synthesized in a 20 *μ*L reverse transcription reaction containing random hexamers and SuperScript III reverse transcriptase (Invitrogen). All real-time PCR reactions were carried out with Bio-Rad iCycler (iQ5 system). The transcript level of* msrA1* was quantified using primers P13 and P14, that of* msrB* was quantified using P15 and P16, and that of the gene encoding the IIa(PTS) was quantified using primers P17 and P18. Transcript levels of genes were normalized to DNA gyrase mRNA using primers P19 and P20 based on a previous report [15, 16]. Changes in gene expression were calculated using the formula 2^−ΔΔCq^ as described [17].

### 2.7. Statistical Analysis

All results are reported as the mean ± SE of at least three independent experiments. Data were analyzed with Student's *t*-test using R Studio for Windows (version 0.98.1103, 3.1.3). Statistical significance was set at *p* ≤ 0.05.

## 3. Results

### 3.1. Construction of* msrA1/msrB* Promoter-*lacZ* Reporter in Wild-Type and* msrA1*,* msrB*,* msrA1-msrB*, and* sigB* Mutants of* S. aureus*

Previously created* msrA1*,* msrB*,* msrA1-msrB*, and* sigB* knockout mutants of* S. aureus* strain SH1000 [6, 9, 10] were transduced in the methicillin-resistant* S. aureus* strain COL. These mutants and the presence of* mecA* gene in these strains were verified by PCR (see Supplemental Figures S1-S2 in Supplementary Material available online at http://dx.doi.org/10.1155/2015/617925). The* msrA1/msrB* promoter-*lacZ* fusion was subsequently integrated into the chromosome of these mutant strains using a bacteriophage transduction procedure. Overall, five* msrA1/msrB* promoter-*lacZ* reporter strains were created in methicillin-resistant as well as methicillin-sensitive* S. aureus* backgrounds. Proper integration of the* msrA1/msrB* promoter-*lacZ* fusion was also confirmed by PCR (Supplemental Figure S3).

### 3.2. Regulation of* msrA1/msrB* Locus in* S. aureus*

**Figure 1:**
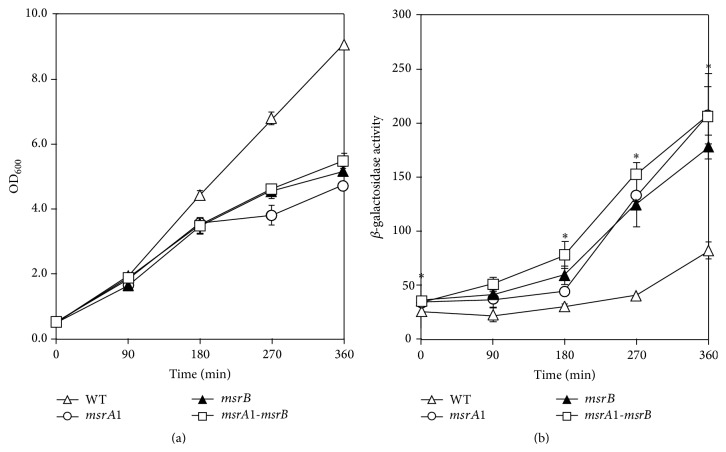
Regulation of* msrA1/msrB* locus in a methicillin-sensitive* S. aureus* strain SH1000. The* msrA1/msrB* promoter-*lacZ* reporter strains were cultured in TSB and growth was measured as OD_600_ (a). *β*-galactosidase activity levels were measured in wild-type* S. aureus* strain SH1000 (open triangles) and its derivatives* msrA1* (open circles),* msrB* (closed triangles), and* msrA1-msrB* (open square) mutants during different stages of growth (b). Values indicate averages of data from at least three independent experiments ± standard error (SE) (*∗* significant at *p* ≤ 0.05).

**Figure 2:**
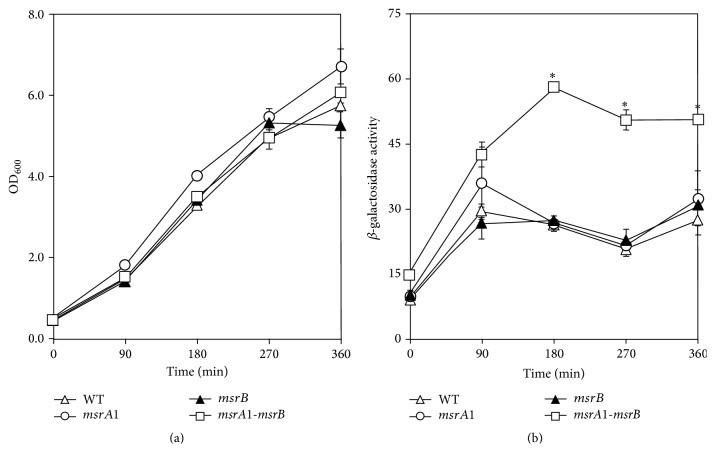
Regulation of* msrA1/msrB* locus in a methicillin-resistant* S. aureus* strain COL. The* msrA1/msrB* promoter-*lacZ* reporter strains were cultured in TSB and growth was measured as OD_600_ (a). *β*-galactosidase activity levels were measured in wild-type* S. aureus* strain COL (open triangles) and its derivatives* msrA1* (open circles),* msrB* (closed triangles), and* msrA1-msrB* (open square) mutants during different stages of growth (b). Values indicate averages of data from at least three independent experiments ± standard error (*∗* significant at *p* ≤ 0.05).

Previously, we reported higher MsrB levels in MsrA1-deficient* S. aureus* cells [9, 10]. This led to the speculation that the* msrA1/msrB* locus may in part be regulated by the products of this locus. To investigate this possibility, the level of *β*-galactosidase was measured in MsrA1-, MsrB-, and MsrA1-MsrB-deficient strains of* S. aureus*. *β*-galactosidase activity levels were higher in these strains compared to the activity level in the wild-type* S. aureus* strain SH1000 (Figure 1). The* msrA1/msrB* promoter-*lacZ* reporter was also studied in the methicillin-resistant strain COL. Overall, the expression of* lacZ* was lower in methicillin-resistant* S. aureus* compared to the methicillin-sensitive* S. aureus* (Figures 1(b) and 2(b)). In addition, *β*-galactosidase activity comparison revealed that only the* msrA1-msrB* double mutant strains had higher activity levels compared to the wild-type COL at the various time points (Figure 2(b)). In the individual* msrA1* or* msrB* mutant strains, a significant increase in *β*-galactosidase activity was not observed compared to wild-type* S. aureus* COL (Figure 2(b)).

### 3.3. Role of SigB in the Regulation of* msrA1/msrB* Locus in* S. aureus*

**Figure 3:**
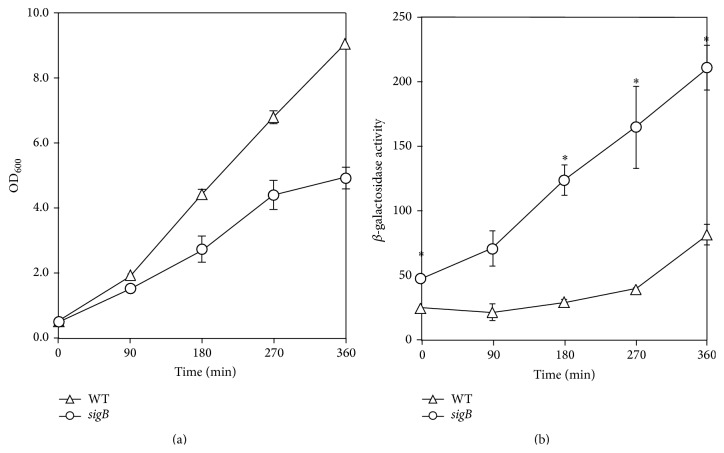
Regulation of* msrA1/msrB locus* in a methicillin-sensitive* S. aureus* strain SH1000 by SigB. The* msrA1/msrB* promoter-*lacZ* reporter strains were cultured in TSB and growth was measured as OD_600_ (a). *β*-galactosidase activity levels were measured in wild-type* S. aureus* strain SH1000 (open triangles) and its derivative* sigB* mutant (closed squares) during different stages of growth (b). Values indicate averages of data from at least three independent experiments ± standard error (*∗* significant at *p* ≤ 0.05).

**Figure 4:**
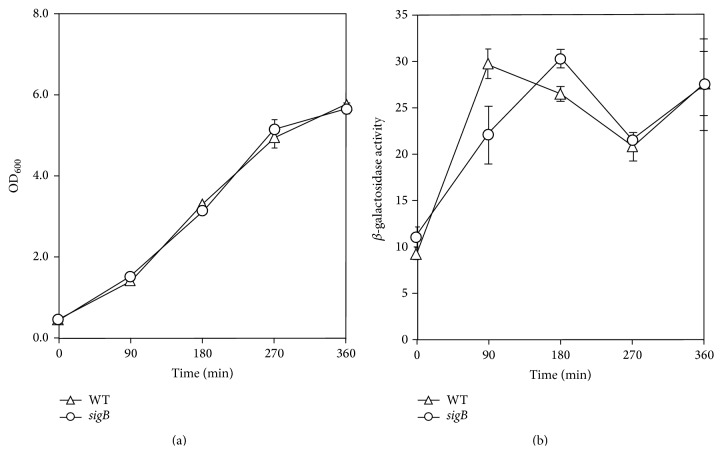
Regulation of* msrA1/msrB* locus in a methicillin-resistant* S. aureus* strain COL by SigB. The* msrA1/msrB* promoter-*lacZ* reporter strains were cultured in TSB and growth was measured as OD_600_ (a). *β*-galactosidase activity levels were measured in* S. aureus* strain COL (open triangles) and its derivative* sigB* mutant (closed squares) during different stages of growth (b). Values indicate averages of data from at least three independent experiments ± standard error (*∗* significant at *p* ≤ 0.05).

Measurement of *β*-galactosidase activity demonstrated that there was increased expression of* lacZ* from the* msrA1/msrB* promoter when* S. aureus* was deficient of SigB in strain SH1000 (Figure 3(b)). However, in* S. aureus* COL, no such increase in the expression of* lacZ* was observed from the* msrA1/msrB *promoter under SigB-deficient conditions compared to the wild-type strain (Figure 4(b)). In qRT-PCR assays, a relatively higher level of* msrA1* transcripts was observed in* sigB* mutant of* S. aureus* strain SH1000 compared to the wild-type strain (Table 3). However, this increase in* msrA1* gene expression was not evident in the* sigB* mutant of* S. aureus* strain COL (Table 3) supporting the findings of the* msrA1/msrB* promoter-*lacZ* data in* sigB* mutant strains.

### 3.4. Induction of* msrA1/msrB* Locus in the Presence of Cell Wall-Active Antibiotic, Oxacillin

**Figure 5:**
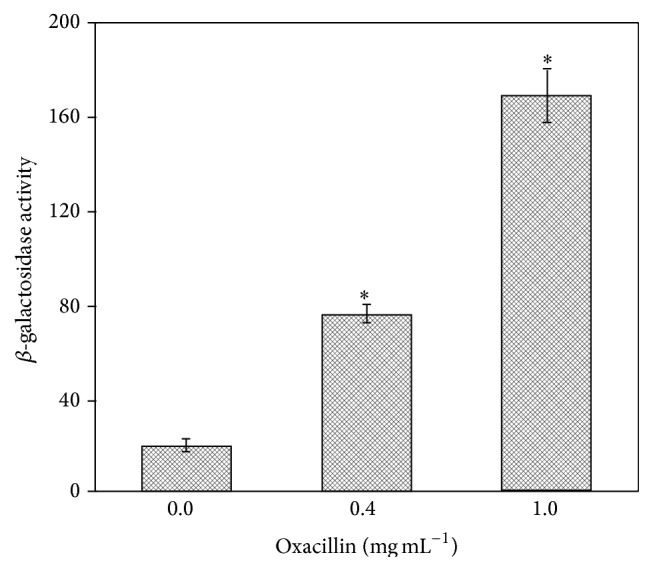
Analysis of* msrA1/msrB* promoter-*lacZ* fusion in* S. aureus* COL in response to oxacillin. Bacterial culture was grown in TSB to OD_600_ = 0.3 and then exposed to oxacillin (0.4 and 1.0 mg mL^−1^, resp.) for 2 h. Subsequently, cells were collected via centrifugation and the *β*-galactosidase activity was determined. Values indicate averages of data from at least three independent experiments ± standard error (*∗* significant at *p* ≤ 0.05).

Previous studies have shown that the* msrA1/msrB* locus is induced by the cell wall-active antibiotics, oxacillin, vancomycin, and D-cycloserine, in a methicillin-sensitive* S. aureus* strain [5]. In a later study, while the* msrA1/msrB* locus remained inducible in the presence of D-cycloserine and vancomycin, no induction of this locus was noted in the presence of oxacillin, when similar experiments were carried out in a methicillin-resistant* S. aureus* strain COL [18]. However, in our experiments, a significantly increased *β*-galactosidase activity clearly indicates a significant induction of* msrA1/msrB* locus in the presence of oxacillin, even in a methicillin-resistant* S. aureus* (Figure 5). We also investigated the expression of the downstream genes of* msrA1* locus in qRT-PCR assays. We determined that the oxacillin stress dramatically induced the expression of* msrA1*,* msrB*, and the gene encoding IIa(PTS) (Table 4). The expression level of the fourth gene of this locus was not investigated due to its very small size. This finding further supports our previous observation of cotranscription of the four genes of the* msrA1/msrB* locus [5, 6].

### 3.5. Expression of* msrA1/msrB* Locus in MsrA1-MsrB-Deficient* S. aureus* Strain COL in the Presence of Oxacillin

**Figure 6:**
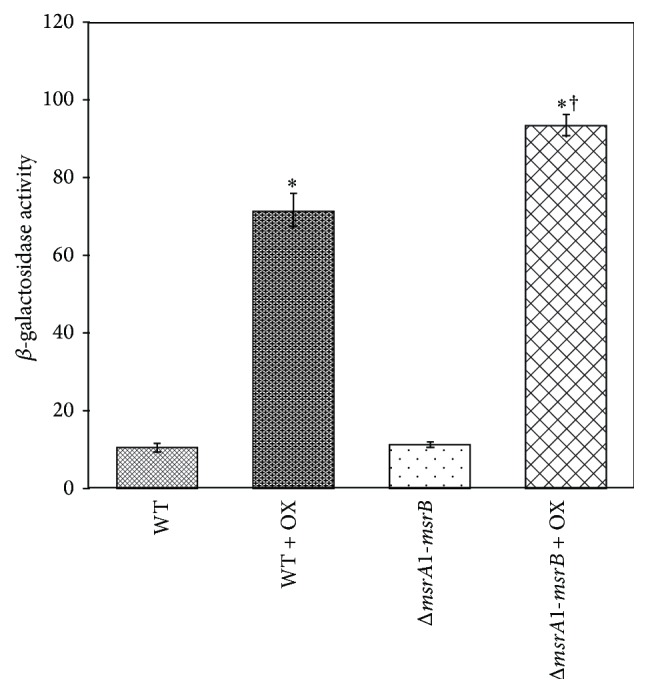
Analysis of* msrA1/msrB* promoter-*lacZ* fusion in wild-type and* msrA1*-*msrB* double mutant of* S. aureus* COL in response to oxacillin. At OD_600_ = 0.5, cells were treated with oxacillin for 2 h and the *β*-galactosidase activity levels were measured. Values indicate averages of data from at least three independent experiments ± standard error (*∗* significant between samples with and without oxacillin at *p* ≤ 0.05; † significant between oxacillin treated wild-type and the oxacillin treated* msrA1-msrB* double mutant at *p* ≤ 0.05).

While studying the regulation of the* msrA1/msrB* locus in a methicillin-resistant* S. aureus* strain COL, oxacillin was added during the growth of the* msrA1/msrB* promoter-*lacZ* reporter to investigate any magnification of the regulation. In these studies, while an increased* lacZ* expression was observed in wild-type* S. aureus* strain COL after oxacillin treatment, a more dramatic increase in the* lacZ* expression in response to oxacillin was seen in MsrA1-MsrB-deficient COL (Figure 6).

## 4. Discussion

Cell wall-active antibiotics have been used extensively for the treatment of infections caused by bacterial pathogens.* S. aureus* is a major human pathogen and is resistant to most commonly available antibiotics. Interestingly, cell wall-active antibiotics cause induction of a locus in* S. aureus* that leads to elevated synthesis of two methionine sulfoxide reductases (MsrA1 and MsrB) [5, 6]. These enzymes reduce methionine sulfoxide and play important roles in maintaining protein integrity and function particularly under oxidative stress. These two proteins have also been shown to have roles in the virulence of bacterial pathogens [19–23]. Msr-deficient bacterial mutants show a reduction in the ability to adhere to eukaryotic cells and are thus less likely to establish an inflection [21, 22, 24, 25]. It is speculated that the lack of the Msr enzymes compromises the integrity of the bacterial surface proteins responsible for adherence to eukaryotic cells. Reduced Msr activity decreases bacterial survival inside the phagocytic cells [20]. In addition to increased levels of MsrA1 and MsrB specifically in response to cell wall-active antibiotics, these proteins in* S. aureus* have been shown to play roles in the survival of bacterial cells under oxidative stress as well as in mice [6, 10].

We previously demonstrated that when the* msrA1* gene is deleted in* S. aureus*, there is an increase in MsrB synthesis suggesting a possible role in the regulation of this locus [9]. Findings of this study suggest that, in a methicillin-sensitive* S. aureus* strain SH1000, MsrA1 and MsrB individually can downregulate the* msrA1/msrB* locus. However, in methicillin-resistant* S. aureus* strain COL, MsrA1 and MsrB both are needed to downregulate the expression of the* msrA1/msrB* locus. It is speculated that the* msrA1/msrB* locus, to some extent, is differentially regulated between methicillin-resistant and methicillin-sensitive* S. aureus* strains. It is not uncommon to observe a differential gene expression pattern between different* S. aureus* strains. It has been demonstrated that the growth of methicillin-resistant* S. aureus* is slower than that of methicillin-sensitive* S. aureus* in the lag phase but not during the exponential phase and that the alterations in virulence between these two strains may at least partially be due to the growth rate differences [26]. Deletion of a gene encoding nitric oxide synthase (NOS) in a methicillin-resistant* S. aureus* reduced virulence as seen by decreased bacterial survival and smaller abscess formation [27]. However, NOS was shown to have a limited role in a methicillin-sensitive* S. aureus* [28]. Expression of genes encoding staphylococcal superantigen-like (SSL) proteins also varies between* S. aureus* strains [29, 30]. Significant differences were also noted between the protein profiles of the methicillin-resistant and methicillin-sensitive* S. aureus* strains exposed to Triton X-100 [31].

It is well established that the* msrA1/msrB* locus is selectively induced in the presence of cell wall-active antibiotics [5]. These antibiotics interfere with the bacterial cell wall synthesis and, as a result, the cells become fragile and susceptible to lysis. Expression of* msrA1/msrB* locus is not induced by antibiotics that target other bacterial metabolic pathways [5]. In a previous report, it was shown that the* msrA1/msrB* locus was not induced by the presence of oxacillin but was induced by the presence of D-cycloserine and vancomycin in a methicillin-resistant* S. aureus* [18]. However, data from our study provide clear evidence that oxacillin does in fact induce the* msrA1/msrB* locus in a methicillin-resistant background of* S. aureus*. The previous report [18] did not observe any induction because the bacterialcells were not exposed to a high enough concentration to impose antibiotic stress in a methicillin-resistant* S. aureus* strain. Furthermore, we explored the induction of* msrA1/msrB* genes in* msrA1*-*msrB* double mutant in methicillin-resistant strain COL. An increase in induction of the* msrA1/msrB* locus was further magnified in* msrA1*-*msrB* double mutant exposed to oxacillin compared to the wild-type* S. aureus* COL in response to oxacillin. This further confirms the notion of downregulation of the* msrA1/msrB* locus by MsrA1 and MsrB and this is more likely an indirect effect. This speculation of an indirect regulation is based on the fact that, after conducting a protein domain search (http://prosite.expasy.org/), no specific DNA-binding domain was observed in MsrA1 and MsrB proteins. It is possible that the MsrA1 and MsrB enzymes are critical in maintaining the integrity of a cytoplasmic transcriptional regulator that is involved in the regulation of expression of this locus.

In recent years, regulation of* msrA* and* msrB* has been studied extensively across multiple species; however, none have shown that MsrA or MsrB directly or indirectly regulates its own expression. It has been demonstrated that RynB regulates the synthesis of* Escherichia coli* MsrB but not MsrA by binding to the 5′ untranslated region of* msrB* mRNA and interfering with its binding to the ribosome [32]. Nitric oxide, which is induced in* Ulva fasciata* upon exposure to light, upregulates the expression of* msr* genes in the intertidal macroalga [33]. In* Saccharomyces cerevisiae*, calcium phospholipid binding protein (CPBP) interacts with the* msrA* promoter and enhances its expression [34]. In* Bacillus subtilis*, a transcriptional regulator, Spx, is shown to significantly upregulate the expression of* msrA* and* msrB* [35]. Spx also upregulates* msrA1* expression in* S. aureus*. Teicoplanin induces* msrA1/msrB* expression in* S. aureus*. However, in* S. aureus spx* mutant, teicoplanin exposure resulted in no significant induction of this locus, whereas, in the* spx* mutant strain complemented with the wild-type* spx* gene,* msrA1/msrB* induction in response to teicoplanin exposure was restored [36]. Additionally, in the* spx* mutant, basal* msrA1* mRNA was significantly lower than* spx* complemented strain [36].

SigB is the alternative sigma factor in* S. aureus* that plays a role in the regulation of expression of stress responsive genes in* S. aureus* [11]. In addition, SigB is also associated with the regulation of expression of the virulence genes in* S. aureus* [11]. In a previous report, the level of expression of* msrA1/msrB* locus was investigated between RN450 (SigB^−^) and SH1000 (SigB^+^) [18]. It was shown that, in* S. aureus* strain SH1000,* msrA1/msrB* expression was 30% more induced than in* S. aureus* strain RN450 in the presence of oxacillin [18]. In contrast, our study shows that SigB in fact downregulates the expression of* msrA1/msrB* locus in* S. aureus* in the methicillin-sensitive* S. aureus* strain SH1000 and plays no role in the regulation of this locus in methicillin-resistant strain COL.

In summary, this study provides evidence that the expression of the* msrA1/msrB* locus is enhanced when* S. aureus* is deficient in MsrA1, MsrB, or both in a methicillin-sensitive* S. aureus*. However, in methicillin-resistant* S. aureus*, increased expression of the* msrA1/msrB* locus was apparent only when the bacterial cells were deficient in both MsrA1 and MsrB. In addition, SigB also in part downregulates the expression of this locus in methicillin-sensitive* S. aureus* but not in methicillin-resistant* S. aureus*.

## Supplementary Material

“The supplemental material contains PCR verification of the bacterial strains used in this study”.

## Figures and Tables

**Table 1:** Bacterial strains used in this study.

Strains	Characteristics	Reference
SH1000	*S*. *aureus* strain 8325-4 with functional RsbU	[11]
COL	Homogeneous in methicillin-resistance expression	[37]
SH1000Δ*msrA1*	*msrA1* mutant of SH1000	[10]
SH1000Δ*msrB*	*msrB* mutant of SH1000	[10]
SH1000Δ*msrA1-msrB*	*msrA1-msrB* double mutant of SH1000	[10]
SH1000Δ*sigB*	*sigB* mutant of SH1000	[14]
COLΔ*msrA1*	*msrA1* mutant of SH1000	[6]
COLΔ*msrB*	*msrB* mutant of SH1000	This study
COLΔ*msrA1-msrB*	*msrA1-msrB* double mutant of SH1000	This study
COLΔ*sigB*	*sigB* mutant of SH1000	[38]
SH1000-(A1/B)P-*lacZ*	*msrA1/msrB* promoter-*lacZ* fusion in SH1000 (Erm^*R*^)	[13]
SH1000Δ*msrA1*-(A1/B)P-*lacZ*	*msrA1/msrB* promoter-*lacZ* fusion in *msrA1* mutant of SH1000 (Kan^*R*^, Erm^*R*^)	This study
SH1000Δ*msrB*-(A1/B)P-*lacZ*	*msrA1/msrB* promoter-*lacZ* fusion in *msrB* mutant of SH1000 (Kan^*R*^, Erm^*R*^)	This study
SH1000Δ*msrA1-msrB*-(A1/B)P-*lacZ*	*msrA1/msrB* promoter-*lacZ* fusion in *msr1-msrB* mutant of SH1000 (Kan^*R*^, Erm^*R*^)	This study
SH1000Δ*sigB*-(A1/B)P-*lacZ*	*msrA1/msrB* promoter-*lacZ* fusion in *sigB* mutant of SH1000 (Kan^*R*^, Erm^*R*^)	This study
COL-(A1/B)P-*lacZ*	*msrA1/msrB* promoter-*lacZ* fusion in COL (Erm^*R*^)	This study
COLΔ*msrA1*-(A1/B)P-*lacZ*	*msrA1/msrB* promoter-*lacZ* fusion in *msrA1* mutant of COL (Kan^*R*^, Erm^*R*^)	This study
COLΔ*msrB*-(A1/B)P-*lacZ*	*msrA1/msrB* promoter-*lacZ* fusion in *msrB* mutant of COL (Kan^*R*^, Erm^*R*^)	This study
COLΔ*msrA1-msrB*-(A1/B)P-*lacZ*	*msrA1/msrB* promoter-*lacZ* fusion in *msrA1-msrB* mutant of COL (Kan^*R*^, Erm^*R*^)	This study
COLΔ*sigB*-(A1/B)P-*lacZ*	*msrA1/msrB* promoter-*lacZ* fusion in *sigB* mutant of COL (Kan^*R*^, Erm^*R*^)	This study

Kan^*R*^: kanamycin resistant; Erm^*R*^: erythromycin resistant.

**Table 2:** Oligonucleotide primers used in this study.

Oligo	Sequence (5′ → 3′)
P1	GCTAACGTCATTGAATATG
P2	GGAAGTAACCTCTGGATCA
P3	GTTACACAAGAAAACGGCA
P4	TCATCATCGTGTTTTGGG
P5	AGGATGTTTCTGGTGCATGG
P6	GACACAACTTCTCCTTCAGT
P7	CCTTTGAACGGAAGTTTGA
P8	TCTAATAGCAACCCACCT
P9	GCTAACGTCATTGAATATG
P10	GGATGGTTCGGATAATGC
P11	GATTGGGATCATAGCGTCA
P12	CTTCAGAGTTAATGGGACCA
P13	AGGCATCAAGTCAGTCGTATC
P14	GAAGTAACCTCTGGATCAAACG
P15	GGTATGGTAAGAACTGAAGTGC
P16	ATTGCAGCGGAATTGATACAG
P17	TCTCCAATTGCAGGACGTGT
P18	ACACTTCAAATCCTTCACCGTCT
P19	TCCACAAGTCGCACGTACAG
P20	GGAAGGCTTGCTACATCTAACG

**Table 3:** Expression levels of *msrA1* in *sigB* mutants relative to wild-type *S*. *aureus* strains SH1000 and COL.

Strain	Fold increase in expression	
	90 min	6 h
SH1000Δ*sigB*1.30	3.16	
COLΔ*sigB*	0.98	1.52

Values indicate averages of three independent experiments.

**Table 4:** Induced expression of *msrA1/msrB* locus genes in *S. aureus* strain COL under oxacillin stress.

Gene	Fold increase in expression under oxacillin stress
*msrA1*	22.9
*msrB*	18.97
*IIa(PTS)*	13.45

Values indicate averages of three independent experiments.
