# Supplementary material for: Regulation of Expression of Oxacillin-Inducible Methionine Sulfoxide Reductases in Staphylococcus aureus
Source: Int J Microbiol. 2015 Sep 21;2015:617925. doi: 10.1155/2015/617925 (PMC4592908; doi:10.1155/2015/617925)
Supplement: Supplementary file 1 — “The supplemental material contains PCR verification of the bacterial strains used in this study”. [file 617925.f1.docx]

**FIGURE LEGENDS (SUPPLEMENTAL FIGURES)**

**Fig. S1. Confirmation of mutations in *S. aureus*.** The mutants were verified by PCR. Primers P1 and P2 were used for the verification of mutation in the *msrA1* gene (lanes 1, 2, 9, 10), P3 and P4 for mutation in the *msrB* (lanes 3, 4, 11, 12), P5 and P6 for mutation in the *msrA1*-*msrB* (lanes 5, 6, 13, 14), and P7 and P8 for mutation in the *sigB* gene (lanes 7, 8, 15, 16), respectively. A larger amplicon (even numbered lanes) was observed when genomic DNA from mutant bacteria was used as template due to insertion of larger antibiotic cassettes in the coding regions compared to smaller amplicons when wild type genomic DNA was used a template (odd numbered lanes).

**Figure S2. Confirmation of presence of *mecA*.** The presence/absence of the *mecA* gene in the various strains was investigated by PCR using primers P9 and P10. The *mecA* gene was present in the wild-type COL (lane 1) and its isogenic *msrA1* (lane 2), *msrB* (lane 3), *msrA1*-*msrB* (lane 4), and *sigB* (lane 5) mutants but absent in the wild-type SH1000 (lane 6) and its isogenic *msrA1* (lane 7), *msrB* (lane 8), *msrA1*-*msrB* (lane 9), and *sigB* (lane 10) mutants.

**Fig. S3. Confirmation of *msrA1/msrB* promoter-*lacZ* integration into *S. aureus*.** A forward primer (P11) internal to the *msrA1/msrB* promoter and a backward primer (P12) internal to the *lacZ* gene were used in the PCR verification of this integration into wild-type (lanes 1 & 6) and isogenic *msrA1* (lanes 2 & 7), *msrB* (lanes 3 & 8), *msrA1- msrB* (lanes 4 & 9), and *sigB* (lanes 5 & 10) mutants.

**Fig. S1**


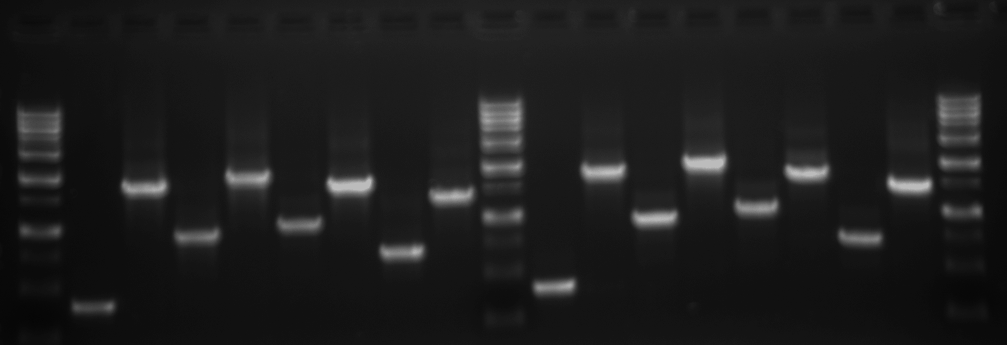


**M**

**2**

**3**

**4**

**5**

**6**

**7**

**8**

**M**

**9**

**10**

**11**

**12**

**13**

**14**

**15**

**16**

**M**

**1**

**8000**

**3000**

**2000**

**1500**

**1000**

**750**

**500**

COL

SH1000

**Fig. S2**


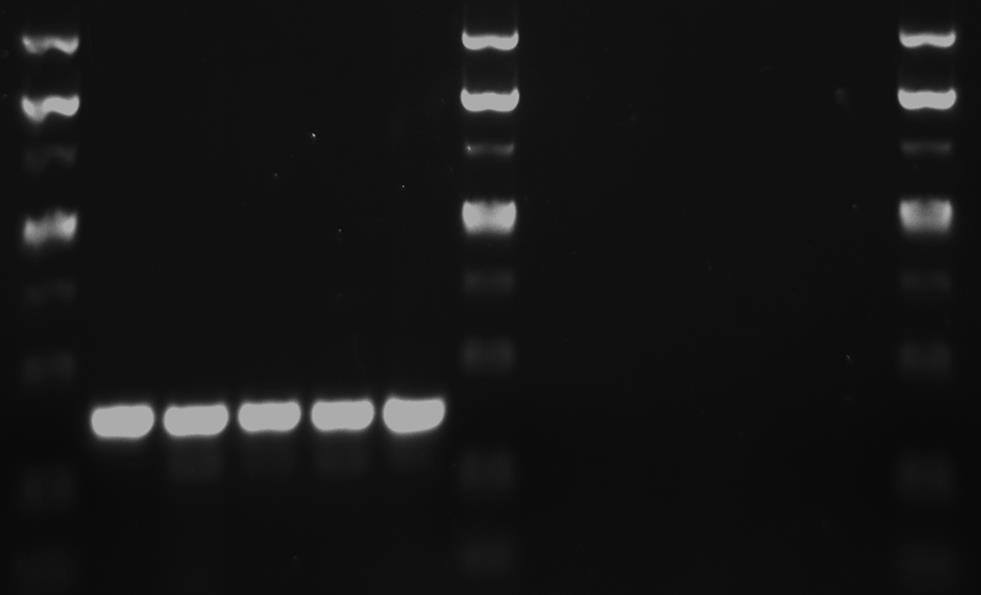


**M**

**2**

**3**

**4**

**5**

**6**

**7**

**8**

**M**

**9**

**10**

**M**

**1**

**4000**

**3000**

**2000**

**1500**

**1000**

**500**

**250**

COL

SH1000

**Fig. S3**


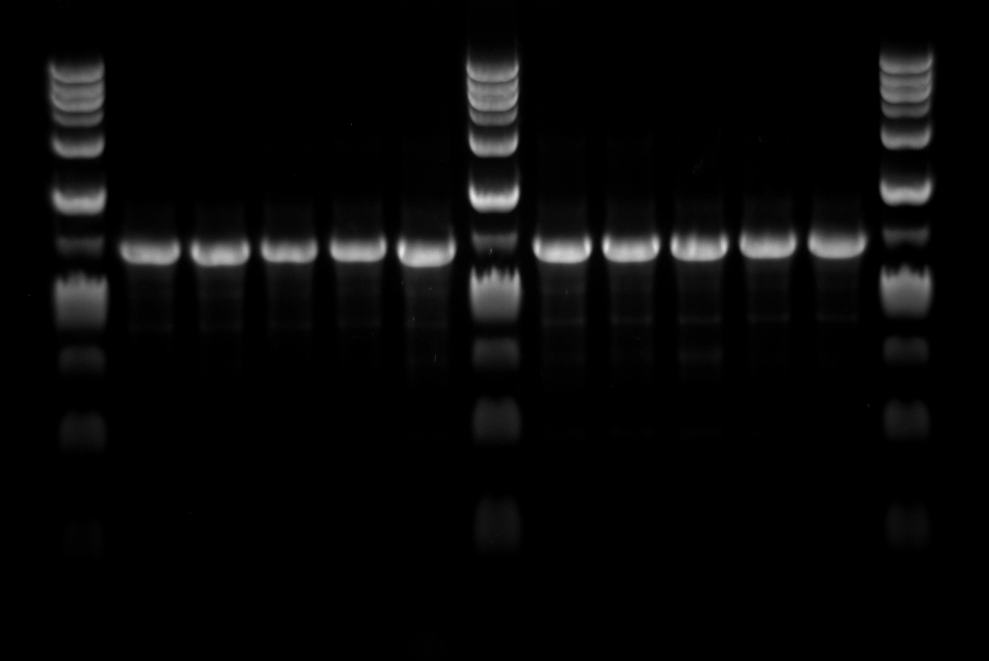


**M**

**2**

**3**

**4**

**5**

**M**

**6**

**7**

**8**

**9**

**10**

**M**

**1**

**8000**

**3000**

**2000**

**1500**

**1000**

COL

SH1000
